# Evaluation of the Dietary Intake of Cadmium, Lead and Mercury and Its Relationship with Bone Health among Postmenopausal Women in Spain

**DOI:** 10.3390/ijerph14060564

**Published:** 2017-05-26

**Authors:** Luis M. Puerto-Parejo, Ignacio Aliaga, María L. Canal-Macias, Olga Leal-Hernandez, Raul Roncero-Martín, Sergio Rico-Martín, Jose M. Moran

**Affiliations:** Metabolic Bone Diseases Research Group, Nursing Department, University of Extremadura, 10003 Caceres, Spain; lmpuerto@unex.es (L.M.P.-P.); i.aliaga@pdi.ucm.es (I.A.); luzcanal@unex.es (M.L.C.-M.); olgaleal@unex.es (O.L.-H.); rronmar@unex.es (R.R.-M.); sergiorico@unex.es (S.R.-M.)

**Keywords:** diet records, heavy metals, quantitative bone ultrasound, dual X-ray absorptiometry, tomography

## Abstract

Background: Heavy metals, such as lead, cadmium, and mercury, are absorbed through contaminated food sources and water. Few studies have investigated the extent to which dietary heavy metals are associated with low bone mineral density. Aims: We aimed to characterize the dietary intake of the heavy metals lead, cadmium and mercury among healthy, non-smoking postmenopausal women in Spain. Furthermore, we sought to establish a putative relationship between bone health and the intake of these heavy metals in this population. Study Design: The daily intake of the heavy metals considered for the different food groups was calculated by accounting for food content and consumption in 281 postmenopausal women. Bone measurements were performed using a Quantitative Bone Ultrasound (QUS), a Dual-Energy X-ray Absorptiometry (DXA) and a Peripheral Quantitative Computed Tomography (pQCT). Results: The average estimated dietary cadmium exposure among the 281 women studied was 29.87 μg/day (20.41–41.04) and 3.03 μg/kg body weight (b.w.; 2.17–4.40). Dietary lead exposure was 46.24 μg/day (38.11–54.77) and 4.87 μg/kg b.w. (4.00–6.14). The estimated dietary mercury exposure was 11.64 μg/day and 1.19 μg/kg b.w. (0.82–1.76). Participants were classified according to their heavy metal intake (above or below the respective medians). After further adjustment for potential confounding factors, no significant differences were found in all the measured parameters (*p* > 0.05). Conclusions: We did not find associations between bone health and the dietary intake of three heavy metals in postmenopausal women. Dietary intake of the measured heavy metals were within the recommended values.

## 1. Introduction

Exposure to heavy metals, such as lead, cadmium, and mercury, occurs through contaminated food sources and water; furthermore, they can be inhaled from cigarette smoke and other sources of air pollution [1]. Cadmium exposure among non-occupationally exposed people occurs primarily via smoking tobacco and secondarily by eating foods containing cadmium [2]. Once inhaled or ingested, cadmium, lead, and mercury are distributed in different tissues and organs [3,4]. Cadmium is inefficiently excreted and accumulates primarily in the liver and kidneys [2]. Tissue stores of cadmium and mercury are slowly excreted from the body via urine and feces at an approximately equal rate, whereas lead is rapidly excreted via urine [3,5].

Cadmium affects the absorption of key divalent cations for bone metabolism such as calcium [1]. There have also been relationships described between several essential elements such as Ca, Fe and Zn that affect their absorption, excretion and tissue retention [1]. Cadmium is also able to interfere with the parathyroid hormone stimulation of vitamin D activation in kidney cells, to increase urinary exretion of Ca, reduce its absorption from the intestines, and to interfere with Ca incorporation into bone cells [6]. Dermience et al. [7] recently reviewed and summarized the toxic effects of lead and cadmium on bone metabolism; their study also highlighted the currently unknown effect of mercury on human bone metabolism and the need for further investigation about the possible effects of mercury on bone metabolism. Lead has been related to increased bone turnover and reduced mineralization, a decrease in bone mineral density (BMD) and mass as well as a cause of osteoporosis in the most severe cases [7]. Lead (Pb^2+^) can substitute to Ca (2+) in hydroxyapatite crystal and additionally lead has a higher affinity for osteocalcin than calcium [7,8]. Cadmium has been associated with a decrease in BMD, cadmium interacts with calcium metabolism and causes hypercalciuria, increased risk of fracture, osteomalacia and osteoporosis and chronic cadmium exposure causes Itai-itai disease [7,9], which is associated with weak and brittle bones. There is also evidence that cadmium disturbs calcium metabolism and calciotropic homones; cadmium decreases liver concentration of other elements such as iron, magnesium, and selenium, and increases levels of copper, zinc and manganese [7].

Previous studies have shown that femur T-scores are associated with the accumulation of cadmium, and this association is gender-specific [5,10,11,12]. Urine cadmium levels have also been associated with osteoporosis [13,14] and dietary intake [15,16]. Even low-level cadmium exposure from food has been associated with low BMD and an increased risk of osteoporosis and fractures [17]. Blood levels of lead, mercury and cadmium are negatively associated with BMD, and this association is gender-specific [18].

Few studies have investigated the extent to which dietary heavy metals are associated with low BMD [17].

We aimed to characterize the dietary intake of the heavy metals lead, cadmium and mercury among healthy, non-smoking postmenopausal women (the population stratum with high cadmium retention) [19] in Spain. Furthermore, we sought to establish a putative relationship between bone health and the intake of these heavy metals in similar groups of women. 

## 2. Materials and Methods

### 2.1. Participants

Healthy postmenopausal women were recruited from the local area via internet advertising and primary care consults. To be eligible for this study, all women had to be healthy, reside in the community, be of white European origin and have no mental or physical functional impairments.

The University of Extremadura Ethical Advisory Committee approved this study. All participants provided written informed consent in accordance with the 1975 Declaration of Helsinki.

We aimed to have enough power to detect a clinically significant 5.4% change in lumbar spine BMD [20]. A sample size of at least *n* = 228 (two groups of 114) was required [21] to achieve a statistical power of 80% and *p* < 0.05. A total of 281 postmenopausal women were included in this study.

All of the women resided in the urban area of the health district of Caceres, Spain. These women underwent primary or secondary examinations. Most of them were married and had children, and their social status was average. None of the participants had dietary restrictions, neurological impairments, or physical disabilities, and their medical histories showed no presence of low-trauma fractures.

We recorded participants’ complete medical histories and physically examined each woman before enrollment in the study. None of the women were taking medications that could interfere with calcium metabolism (e.g., corticoids, oral anticoagulants, antipsychotics, etc.). All of the women led active lives but did not regularly exercise. Alcohol intake was sporadic and did not exceed 100 mL/day in any case. None of the women smoked. Height was measured using a Harpenden stadiometer with a mandible plane parallel to the floor, and weight was measured using a biomedical precision balance scale. Both measurements were determined when the participants were wearing only light clothing and no shoes. Body mass index (BMI) was calculated as the weight in kilograms divided by the square of the height in meters (kg/m^2^).

### 2.2. Bone Measurements

An ultrasound was performed on the 2nd to the 5th proximal phalanx of the non-dominant hand using a DBM Sonic Bone Profiler (IGEA, Capri, Italy).

The femoral neck and L2–L4 spine BMDs were measured via dual-energy X-ray absorptiometry DXA (Norland XR-800, Norland Inc., Fort Atkinson, WI, USA) and expressed as the quantity of mineral (g) divided by the area scanned (cm^2^).

pQCT measurements were performed on the non-dominant distal forearm using a Stratec XCT-2000 device (Stratec Medizintechnik, Pforzheim, Germany).

### 2.3. Assessment of Diet and Covariates

According to Food and Agriculture Organization/World Health Organization (FAO/WHO) recommendations (WHO, 1985), three basic approaches are employed to assess the intake of food contaminants or other dietary elements: (a) total diet studies (TDSs), (b) duplicate diet studies, and (c) diary studies that combine the data for specific contaminants with individual (or household) consumption records (Perello et al., 2014). Women enrolled in this study completed a 131-item food frequency questionnaire (FFQ). This FFQ was previously validated and involves 24-h recall performed over seven days [22,23,24,25,26]. A food cadmium, lead and mercury database was constructed based on the cadmium, lead and mercury contents previously reported with regard to the Spanish market [27]. Significant differences were not observed in the dietary patterns across geographical areas of Spain; however, the quantities consumed differed greatly [28]. The daily intake of the elements considered for the different food groups was calculated by accounting for food content and consumption. The toxic element concentrations of the different food groups were taken from the literature [28]. Using the FFQ, we also assessed the dietary intake of calcium and vitamin D. Information regarding calcium and vitamin D originated from the Spanish Food Composition database [29].

### 2.4. Statistical Analyses

Medians and the interquartile ranges were used to describe the sample.

Because of the asymmetric distribution of many of the studied variables (i.e., age, Ad-SoS, FN BMD, WT BMD, L2 BMD, L3 BMD, L2–L4 BMD, total area mm^2^, cortical area mm^2^, daily Cd intake, vitamin D intake, Ca intake, Fe intake, Mg, intake and Kcal intake) and the presence of atypical values (i.e., outliers), the non-parametric Wilcoxon test was used to evaluate the differences between groups with regard to the studied variables. To adjust for potential confounds, we used a non-parametric rank analysis of covariance model, where heavy metal intake was considered a factor, and kcal intake was considered a covariate. All statistical tests were conducted in SPSS version 22.0 (IBM Corp., Armonk, NY, USA).

## 3. Results

### 3.1. Dietary Heavy Metals Exposure and Major Food Sources in the Whole Sample 

The average estimated dietary cadmium exposure among the 281 women studied was 29.87 μg/day (20.55–40.90) and 3.04 μg/kg body weight (b.w.; 2.19–4.41). Dietary lead exposure was 46.25 μg/day (38.13–56.71) and 4.88 μg/kg b.w. (4.01–6.09). The estimated dietary mercury exposure was 11.64 μg/day (7.68–16.10) and 1.19 μg/kg b.w. (0.82–1.77).

**Figure 1:**
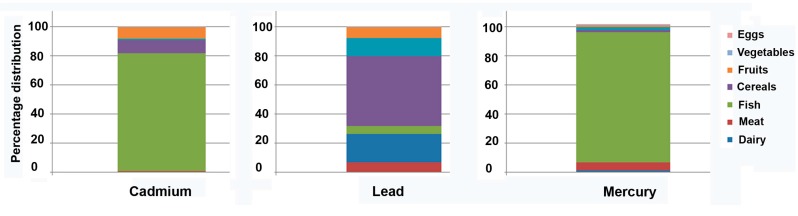
Percentage contribution of various food groups towards the total daily diet.

The major sources of dietary cadmium exposure were fish and cereals, which constituted 89% of the total intake (Figure 1). The major sources of dietary lead were cereals, meat and fruits, together accounting for 80% of the total intake (Figure 1). Finally, the major sources of dietary mercury were fish and meat, constituting 94% of the total intake.

### 3.2. Sample Characteristics with Regard to Low and High Heavy Metal Dietary Exposure (Above or Below the Respective Medians)

Table 1 (cadmium), Table 2 (lead) and Table 3 (mercury) show the sample characteristics with regard to low and high heavy metal dietary exposure (above or below the respective medians). Women with high dietary heavy metal exposure reported consuming more vitamin D, calcium, iron and magnesium than those with less dietary heavy metal exposure. After adjusting for calorie consumption, no differences were found with regard to dietary vitamin D consumption in the lead subgroups or dietary calcium consumption in the mercury subgroups (Table 2 and Table 3, respectively). The remaining studied were also significant. Dietary exposure to heavy metals was positively associated with dietary calcium (r = 0.147; *p* = 0.014), dietary iron (r = 0.380; *p* < 0.001), and dietary magnesium (r = 0.220; *p* < 0.001) but was not associated with the intake of vitamin D (*p* = 0.414) after adjusting for calorie intake. Similarly, dietary lead was positively associated with dietary calcium (r = 0.232; *p* < 0.001), dietary iron (r = 0.421; *p* < 0.001), and dietary magnesium (r = 0.220; *p* < 0.001) but not associated with vitamin D intake (*p* = 0.878). Dietary vitamin D was also not associated with the dietary mercury (*p* = 0.422), whereas dietary calcium was associated with dietary mercury (r = 0.133; *p* = 0.027). Dietary iron (r = 0.372; *p* < 0.001) and dietary magnesium (r = 0.201; *p* < 0.001) were also associated.

### 3.3. Bone Health and Dietary Intake of Heavy Metals

Table 4 (cadmium), Table 5 (lead) and Table 6 (mercury) show the bone density parameter data. A significant difference was only observed in trabecular density, and this difference remained significant after adjusting for calorie intake in the lead subgroup analysis (*p* = 0.049). Women who consumed more lead presented higher trabecular densities than those with less lead intake. A significant difference was also found in L2 BMD, but this difference remained non-significant after adjustment (*p* = 0.056). No other differences were found in the subgroup analysis.

### 3.4. Risk of Low Bone Mineral Density and Dietary Intake of Heavy Metals

We explored the risk of low BMD (i.e., a T-score < −1) at either the hip (femoral neck) or the lumbar spine for dietary cadmium, lead and mercury. We observed a non-significant OR of 0.840 (95% CIs = 0.363–1.944; *p* = 0.68) for the hip and a non-significant OR of 1.386 (95% CIs = 0.766–2.510; *p* = 0.28) for the lumbar spine among the cadmium groups. Similarly, a non-significant OR of 1.008 (95% CIs = 0.437–2.327; *p* = 0.98) was observed for the hip, and a non-significant OR of 1.520 (95% CIs = 0.838–2.759; *p* = 0.15) was observed for the lumbar spine among the lead subgroup. A non-significant OR of 0.84 (95% CIs = 0.363–1.944; *p* = 0.68) was observed for the hip, and a non-significant OR of 1.668 (95% CIs = 0.916–3.038; *p* = 0.09) was observed for the lumbar spine among the dietary mercury subgroup. We also explored the risk of low BMD after adjusting for the dietary intake of calcium, magnesium, iron, vitamin D and calories via a logistic regression. A non-significant OR was observed for the hip (OR = 1.461; 95% CIs = 0.571–3.741; *p* = 0.429) and lumbar spine (OR = 0.767; 95% CIs = 0.396–1.489; *p* = 0.432) among the cadmium subgroup. Furthermore, a non-significant OR was observed for the lumbar spine (OR = 0.830; 95% CIs = 0.399–1.729; *p* = 0.031) and the hip (OR = 1.221; 95% CIs = 0.429–3.469; *p* = 0.709) among the lead subgroup. Finally, no significant differences were observed in the mercury subgroup for the hip (OR = 1.458; 95% CIs = 0.557–3.818; *p* = 0.443) or lumbar spine (OR = 0.652; 95% CIs = 0.344–1.237; *p* = 0.191).

### 3.5. Combined Intake of Heavy Metals and Bone Health

In the final analysis, we assessed the combined effect of a high dietary intake of the studied heavy metals. These data are shown in Table 7. No significant differences were found between the groups for age (*p* = 0.593) or BMI (0.052). Significant differences were found regarding L2 BMD (*p* = 0.013) and the lumbar spine (*p* = 0.023). In both cases, BMD was slightly higher in women with a higher intake of heavy metals. The differences regarding the lumbar spine remained after adjusting for calorie intake (*p* = 0.037). The differences observed in the trabecular area (*p* = 0.004) remained non-significant after adjusting for calorie intake (*p* = 0.107).

## 4. Discussion

Our sample of postmenopausal women had a dietary cadmium intake that was notably lower than the provisional tolerable weekly intake (PTWI) established by the Joint FAO/WHO Expert Committee on Food Additives (JECFA; 7 μg/kg b.w./week) [30]. In 2009, however, the European Food Safety Authority (EFSA) [31,32] reevaluated data on dietary cadmium intake and set a new PTWI of 2.5 μg/kg b.w. (0.357 μg/kg b.w./day) [33]. Approximately 66% of the sample exceeded the threshold for this element. 

JECFA has established a PTWI for lead of 25 μg/kg b.w.; however, the EFSA [34] concluded that the former PTWI (given as μg/kg b.w.) was not appropriate because no evidence of a critical threshold for lead-induced effects exists. In 2011, the JECFA concluded that because of prior analyses, a critical threshold would be considered health protective [33]. Nevertheless, all of the women had values well below the PTWI. 

With respect to mercury, the EFSA recommends a maximum intake of 4 µg/kg b.w./week [33], and only 0.7% of the sample exceeded this threshold. Previous studies [27,35] in different areas of Spain studied the intake of heavy metals and other potentially toxic materials. The results obtained regarding the average dietary intake of lead ranged from 4 µg/kg b.w./week to 56 µg/kg b.w./week among the four areas studied. These results are similar to the dietary intakes assessed in our area (4.8 µg/kg b.w./week).

In 2014, the temporal trends of the dietary intakes of cadmium, lead and mercury (2000, 2005, 2008 and 2012) were estimated for Catalonia, Spain [33]. The dietary intakes for cadmium, mercury, and lead in this region were 0.87 µg/kg b.w./week, 1.1 µg/kg b.w./week and 0.84 µg/kg b.w./week, respectively; these figures are below the values observed in our sample. The data from our study confirm previous studies in different areas of Spain showing that the dietary intakes of heavy metals in the Spanish diet are generally within the recommended limits [36,37,38,39,40,41,42,43]. Moreiras and Cuadrado [28] examined the estimated intake of heavy metals in the diets of people from our specific area of Spain in 1992 and indicated that Extremadura showed the theoretically lowest intake of cadmium but the highest intake of mercury in all of Spain. It is possible that the dietary habits of our area have changed since then, thereby changing the trend in the dietary intake of the studied heavy metals.

According to Perelló and colleagues [33], the major dietary sources of cadmium are cereals and fish; these conclusions corroborate our results. Similar results were found for the intake of mercury: fish was the major dietary contributor. Cereals made the greatest contribution to total dietary lead intake in three of the four areas studied; this result supports our observation about the contribution of cereals to the dietary intake of lead in the current sample. A previous study also showed that cereals are a major source of dietary lead and cadmium in Spain [44].

An association exists between dietary cadmium exposure and higher rates of bone fracture (including hip fracture) independent of tobacco smoking in men [45]. Similarly, even low-level exposure to dietary cadmium has been associated with bone fragility among postmenopausal women [17]. Recently, positive associations with osteoporosis-related incident fractures were described in a cohort of elderly Swedish men, and these associations were also described in men who had never smoked but who were exposed primarily through their diets [46]. These results show that older men with relatively low dietary cadmium exposure are also at an increased risk of low BMD and fracture associated with cadmium.

Although high cadmium exposure causes bone damage, the association between low-level cadmium exposure (i.e., dietary exposure) and bone health must be clarified, especially in women [47]. Little evidence exists on the associations between BMD status and cadmium, lead or mercury intake among osteopenic or osteoporotic Korean adults [48]. However, the negative effects of low-level cadmium exposure on bone, possibly exerted via increased bone resorption, seem to intensify after menopause [47]. We did not observe any association between BMD and dietary cadmium intake in our cohort of Spanish women. Recent research has focused on the putative roles that cadmium plays in volumetric BMD and bone morphometry. In vivo studies of Sprague-Dawley male rats have shown that cadmium exposure can induce low vBMD [49] by decreasing the trabecular number, thereby reducing the quantity of mineralized bone tissue.

Similarly, conflicting evidence suggests that bone lead or blood lead reduces areal BMD. Recent data have shown that bone lead accumulated from exposure over time can detrimentally affect bone by reducing cortical thickness and integral volumetric bone density [50] in postmenopausal women. We observed a non-significant difference in the trabecular density between the groups of women with either high or low dietary intakes of lead; the trabecular density of the higher dietary cadmium intake group was greater. We believe that this result deserves additional study because it might support the hypothesis that lead affects volumetric BMD.

The strengths of our study include its use of a heavy metals database based on concentrations in foods sold on the Spanish market as well as the assessment of BMD via DXA (the reference standard for BMD measurements), volumetric BMD and bone morphometry via pQCT and bone quality via QUS.

We recognize that our study has several limitations. First, its cross-sectional design does not allow us to establish causal relationships. Second, although we adjusted for calorie intake and no differences were observed in the major determinants of bone density between the studied groups (age, BMI, calcium intake, vitamin D intake, or smoking status), we cannot completely exclude the possibility that our findings are biased by unmeasured or residual confounds. Additionally, we aimed to have enough power to detect a least significant change in spine BMD of 5.4% that was established for intragroup comparisons and this could be conservative. Therefore, our study might have a moderate risk of finding no difference when in fact a difference might exist below the indicated threshold. Finally, dietary estimation of cadmium exposure should be used with caution in our study because of the lack of association between estimated dietary cadmium and measured urinary cadmium exposure. 

## 5. Conclusions

The current study provides the first observational data regarding volumetric BMD, bone morphometry, QUS and the dietary intakes of cadmium, lead and mercury among Spanish women. With the use of newer imaging techniques, such as pQCT, which provide more information about bone quality than standard methods such as DXA, we did not find associations between bone health and the dietary intakes of three heavy metals.

## Figures and Tables

**Table 1:** Sample characteristics of low and high cadmium dietary exposure (above or below the respective medians).

	Low (<29.87 µg/day)	High (>29.87 µg/day)	*p*-Value	*p*-Value *
	Median (IQR)	Median (IQR)		
Age at measurement	57 (54–61)	59 (55–63)	0.18	
Body mass index (kg/m2)	27.71 (24.63–30.41)	27.24 (24.33–28.99)	0.11	
Dietary vitamin D (µg/day)	4.08 (2.47–7.15)	8.67 (4.89–13.33)	<0.001	<0.001
Dietary calcium (mg/day)	915 (613–1221.5)	1214 (883–1528)	<0.001	0.04
Dietary Iron (mg/day)	12.01 (9.5–15.41)	15.91 (13.28–21.88)	<0.001	<0.001
Dietary energy (Kcal/day)	1996.7 (1645.7–2317.1)	2360.05 (1946.97–2712.27)	<0.001	N/A
Dietary magnesium (mg/day)	221 (173.4–292.3)	322.2 (234.95–407.57)	<0.001	<0.001
Dietary cadmium/body weigth (µg/kg b.w)	2.18 (1.61–2.66)	4.40 (3.65–5.85)	<0.001	<0.001
Dietary cadmium (µg/day)	20.55 (15.60–25.10)	41.04 (34.90–54.55)	<0.001	<0.001

* After further adjustment by energy intake.

**Table 2:** Sample characteristics of low and high lead dietary exposure (above or below the respective medians).

Characteristics	Low (<46.25 µg/day)	High (>46.5 µg/day)	*p*-Value	*p*-Value *
	Median (IQR)	Median (IQR)		
Age at measurement	58 (54.5–63)	58 (55–62)	0.74	
Body mass index (kg/m^2^)	27.77 (24.61–30.32)	27.32 (24.25–29.09)	0.08	
Dietary vitamin D (µg/day)	4.04 (2.46–8.5)	7.65 (4.53–12.01)	<0.001	0.07
Dietary calcium (mg/day)	798 (586–1105)	1293 (1005–1589.25)	<0.001	<0.001
Dietary Iron (mg/day)	11.35 (8.96–14.17)	16.99 (13.99–22.47)	<0.001	<0.001
Dietary energy (Kcal/day)	1882.3 (1546.65–2209.9)	2437.2 (2164.97–2778.05)	<0.001	N/A
Dietary magnesium (mg/day)	201.5 (168.85–270.55)	339.95 (258.92–427.72)	<0.001	<0.001
Dietary lead/body weigth (µg/kg b.w)	4.01 (3.34–4.61)	56.77 (50.65–67.95)	<0.001	<0.001
Dietary lead (µg/day)	38.13 (32.96–42.27)	6.14 (5.25–7.84)	<0.001	<0.001

* After further adjustment by energy intake.

**Table 3:** Sample characteristics of low and high mercury dietary exposure (above or below the respective medians).

Characteristics	Low (<11.65 µg/day)	High (>11.65 µg/day)	*p*-Value	*p*-Value *
	Median (IQR)	Median (IQR)		
Age at measurement	57 (54–62)	58.5 (55–63)	0.28	
Body mass index (kg/m^2^)	27.58 (24.61–30.41)	27.33 (24.45–29.09)	0.25	
Dietary vitamin D (µg/day)	3.78 (2.26–7,13)	8.67 (5.08–13.33)	<0.001	<0.001
Dietary calcium (mg/day)	902 (618.5–1232.5)	1208 (896.5–1528)	<0.001	0.12
Dietary Iron (mg/day)	11.74 (9.47–15.12)	16.26 (13.35–22.05)	<0.001	<0.001
Dietary energy (Kcal/day)	1981.2 (1610.65–2305.1)	2362.15 (1989.42–2712.27)	<0.001	N/A
Dietary magnesium (mg/day)	215.3 (173.75–290.4)	322.2 (239.3–407.57)	<0.001	<0.001
Dietary mercury/body weigth (µg/kg b.w)	4.19 (3.51–5.01)	53.21 (45.10–66.40)	<0.001	<0.001
Dietary mercury (µg/day)	40.05 (34.24–46.63)	5.80 (4.73–7.36)	<0.001	<0.001

* After further adjustment by energy intake.

**Table 4:** Quantitative bone ultrasound, bone mineral density and volumetric bone mineral density for low and high cadmium dietary exposure (above or below the respective medians).

Measurement	Low (<29.87 µg/day)	High (>29.87 µg/day)	*p*-Value
	Median (IQR)	Median (IQR)	
**Quantitative bone ultrasound**			
Ad-SoS (m/s)	2042 (1995.5–2088)	2040 (2000–2093.25)	0.986
**Bone mineral density (gr/cm^2^)**			
BMD Femoral neck	0.829 (0.769–0.914)	0.839 (0.772–0.901)	0.968
BMD Femoral trochanter	0.684 (0.609–0.733)	0.667 (0.619–0.726)	0.629
BMD Ward’s triangle	0.599 (0.542–0.684)	0.621 (0.545–0.673)	0.657
BMD L2	1.005 (0.937–1.097)	1.000 (0.945–1.065)	0.322
BMD L3	1.044 (0.966–1.125)	1.033 (0.975–1.091)	0.282
BMD L4	1.019 (0.951–1.104)	0.991 (0.928–1.094)	0.098
BMD lumbar spine	1.026 (0.960–1.098)	1.004 (0.950–1.077)	0.172
**Volumetric BMD (mg/cm^3^)**			
Total density	337.5 (306.95–369.9)	337.2 (300.775–367.55)	0.702
Trabecular density	181.4 (162.45–207.2)	173.55 (149.65–203.325)	0.339
Cortical density	458.1 (413.4–508.5)	468.95 (418–513.725)	0.085
**Bone morphometry (mm^2^)**			
Total area	298 (273.75–323.35)	292.45 (265.55–317.1)	0.337
Trabecular area	134 (123.05–145.35)	131.6 (119.7–142.4)	0.608
Cortical area	163.8 (150.45–177.42)	161 (146.3–174.625)	0.351

**Table 5:** Quantitative bone ultrasound, bone mineral density and volumetric bone mineral density for low and high lead dietary exposure (above or below the respective medians).

Measurement	Low (<46.25 µg/day)	High (>46.25 µg/day)	*p*-Value	*p*-Value *
	Mean (IQR)	Mean (IQR)		
**Quantitative bone ultrasound**				
Ad-SoS (m/s)	2037 (1994–2085.5)	2042 (2007.25–2096)	0.13	
**Bone mineral density (gr/cm^2^)**				
BMD Femoral neck	0.829 (0.766–0.913)	0.835 (0.7745–0.898)	0.943	
BMD Femoral trochanter	0.684 (0.608–0.737)	0.669 (0.611–0.717)	0.575	
BMD Ward’s triangle	0.609 (0.542–0.684)	0.616 (0.544–0.679)	0.934	
BMD L2	1.032 (0.952–1.097)	0.990 (0.936–1.053)	**0.008**	0.056
BMD L3	1.05 (0.975–1.125)	1.03 (0.962–1.089)	0.087	
BMD L4	1.015 (0.943–1.104)	0.995 (0.935–1.086)	0.217	
BMD lumbar spine	1.027 (0.964–1.099)	0.999 (0.949–1.07)	0.052	
**Volumetric BMD (mg/cm^3^)**				
Total density	340.2 (307.2–376.9)	334.8 (302.775–362.175)	0.109	
Trabecular density	291.4 (266.8–318.7)	299.7 (273.475–324.325)	**0.001**	**0.049**
Cortical density	186.8 (163.7–210.85)	171.15 (149.65–195.3)	0.746	
**Bone morphometry (mm^2^)**				
Total area	130.9 (120.25–142.95)	135.25 (123.6–145.9)	0.333	
Trabecular area	460.1 (414.35–516.1)	465.6 (417.6–503.875)	0.269	
Cortical area	160.5 (146.975–173.625)	165 (150.975–178.6)	0.242	

* After further adjustment by energy intake.

**Table 6:** Quantitative bone ultrasound, bone mineral density and volumetric bone mineral density for low and high mercury dietary exposure (above or below the respective medians).

Measurement	Low (<11.65 µg/day)	High (>11.65 µg/day)	*p*-Value
	Mean (IQR)	Mean (IQR)	
**Quantitative bone ultrasound**			
Ad-SoS (m/s)	2042 (1997–2088.5)	2040 (1999.25–2089.75)	0.956
**Bone mineral density (gr/cm^2^)**			
BMD Femoral neck	0.829 (0.769–0.913)	0.839 (0.772–0.901)	0.984
BMD Femoral trochanter	0.684 (0.61–0.735)	0.667 (0.612–0.723)	0.554
BMD Ward’s triangle	0.600 (0.543–0.690)	0.62 (0.543–0.670)	0.868
BMD L2	1.003 (0.951–1.097)	1.005 (0.937–1.067)	0.267
BMD L3	1.043 (0.972–1.118)	1.033 (0.974–1.093)	0.341
BMD L4	1.015 (0.951–1.104)	0.996 (0.928–1.094)	0.125
BMD lumbar spine	1.026 (0.961–1.096)	1.006 (0.948–1.084)	0.183
**Volumetric BMD (mg/cm^3^)**			
Total density	338.3 (306.95–369.9)	336.8 (300.775–367.55)	0.655
Trabecular density	294.1 (269.1–323.35)	294.5 (267.475–317.4)	0.084
Cortical density	182.2 (161.5–207.45)	173.55 (152.475–200.725)	0.69
**Bone morphometry (mm^2^)**			
Total area	132.3 (121.85–144.8)	132.8 (120.4–143.225)	0.859
Trabecular area	458.2 (414.1–508.5)	467.9 (417.6–513.725)	0.99
Cortical area	161.7 (149.175–177.1)	162.25 (147.3–174.7)	0.96

**Table 7:** Combined association of a high dietary intake of the studied heavy metals over the quantitative bone ultrasound, bone mineral density and volumetric bone mineral density.

	Combined High Dietary Intake of Heavy Metals	Combined Low Intake of Heavy Metals	*p*-Value
	Median (IQR)	Median (IQR)	
**Quantitative bone ultrasound**			
Ad-SoS (m/s)	2038.5 (1995.25–2086)	2041 (2006.5–2095)	0.424
**Bone mineral density (gr/cm^2^)**			
BMD Femoral neck	0.829 (0.770–0.914)	0.842 (0.782–0.906)	0.766
BMD Femoral trochanter	0.692 (0.609–0.746)	0.676 (0.622–0.730)	0.515
BMD Ward’s triangle	0.602 (0.543–0.687)	0.620 (0.547–0.680)	0.836
BMD L2	1.032 (0.954–1.103)	0.995 (0.941–1.053)	0.013
BMD L3	1.059 (0.977–1.14)	1.034 (0.975–1.091)	0.051
BMD L4	1.026 (0.954–1.124)	0.994 (0.934–1.097)	0.071
BMD lumbar spine	1.042 (0.965–1.106)	1.004 (0.947–1.069)	0.023 *
**Volumetric BMD (mg/cm^3^)**			
Total density	339.25 (310.525–375.7)	335.2 (301.9–366.1)	0.2
Trabecular density	295.7 (271.9–321.95)	301 (273.65–324.25)	0.004
Cortical density	185.05 (162.275–207.2)	168.3 (148.55–193.25)	0.922
**Bone morphometry (mm^2^)**			
Total area	133 (122.2–144.625)	135.4 (123.6–145.9)	0.722
Trabecular area	458.15 (417.575–525.6)	469.6 (418.55–515.2)	0.662
Cortical area	161.9 (149.7–176.8)	165.7 (151.25–178.6)	0.619

* *p* = 0.037 after further adjustment by energy intake.
